# {4,4′,6,6′-Tetra­chloro-2,2′-[2,2-dimethyl­propane-1,3-diylbis(nitrilo­methanylyl­idene)]}copper(II)

**DOI:** 10.1107/S160053681200195X

**Published:** 2012-01-21

**Authors:** Hadi Kargar, Reza Kia, Saeideh Abbasian, Muhammad Nawaz Tahir

**Affiliations:** a Department of Chemistry, Payame Noor University, PO Box 19395-3697 Tehran, I. R. of IRAN; b X-ray Crystallography Lab., Plasma Physics Research Center, Science and Research Branch, Islamic Azad University, Tehran, Iran; c Department of Chemistry, Science and Research Branch, Islamic Azad University, Tehran, Iran; d Department of Physics, University of Sargodha, Punjab, Pakistan

## Abstract

In the title Schiff base complex, [Cu(C_19_H_16_Cl_4_N_2_O_2_)], the geometry around the Cu^II^ atom is distorted square-planar defined by the N_2_O_2_ donor atoms of the coordinated ligand. The dihedral angle between the substituted benzene rings is 29.95 (16)°. In the crystal, mol­ecules are linked along the *b* axis, forming individual dimers through C—H⋯O inter­actions. The crystal structure is further stabilized by inter­molecular π–π inter­actions [centroid–centroid distance = 3.6131 (17) Å].

## Related literature

For standard values of bond lengths, see: Allen *et al.* (1987 ▶). For applications of Schiff bases in coordination chemistry, see, for example: Granovski *et al.* (1993 ▶); Blower (1998 ▶). For related structures see, for example: Ghaemi *et al.* (2011 ▶); Kargar *et al.* (2011 ▶, 2012 ▶).
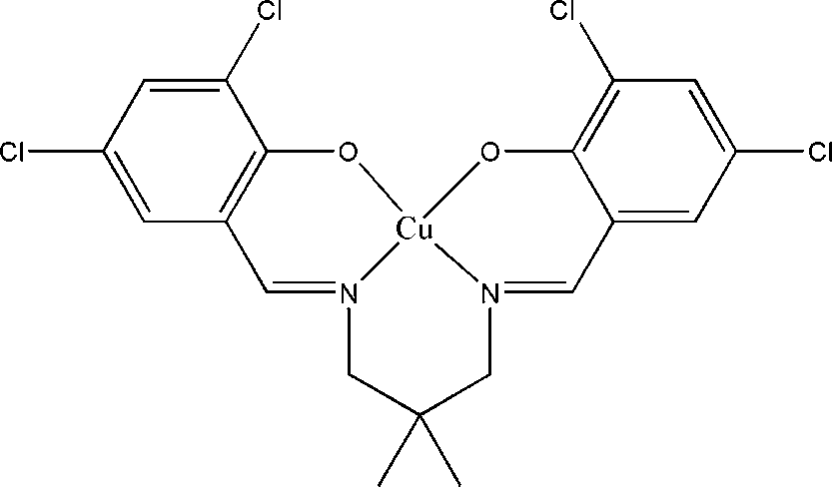


## Experimental

### 

#### Crystal data

[Cu(C_19_H_16_Cl_4_N_2_O_2_)]
*M* *_r_* = 509.68
Monoclinic, 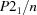

*a* = 12.4002 (10) Å
*b* = 8.4570 (7) Å
*c* = 20.0316 (19) Å
β = 97.278 (4)°
*V* = 2083.8 (3) Å^3^
*Z* = 4
Mo *K*α radiation
μ = 1.58 mm^−1^
*T* = 291 K
0.25 × 0.18 × 0.09 mm

#### Data collection

Bruker SMART APEXII CCD area-detector diffractometer
Absorption correction: multi-scan (*SADABS*; Bruker, 2005 ▶) *T*_min_ = 0.694, *T*_max_ = 0.871
18291 measured reflections
4988 independent reflections
2882 reflections with *I* > 2σ(*I*)
*R*_int_ = 0.060

#### Refinement

*R*[*F* ^2^ > 2σ(*F* ^2^)] = 0.047
*wR*(*F* ^2^) = 0.105
*S* = 1.00
4988 reflections
255 parameters
H-atom parameters constrained
Δρ_max_ = 0.38 e Å^−3^
Δρ_min_ = −0.36 e Å^−3^

### 

Data collection: *APEX2* (Bruker, 2005 ▶); cell refinement: *SAINT* (Bruker, 2005 ▶); data reduction: *SAINT*; program(s) used to solve structure: *SHELXTL* (Sheldrick, 2008 ▶); program(s) used to refine structure: *SHELXTL*; molecular graphics: *SHELXTL*; software used to prepare material for publication: *SHELXTL* and *PLATON* (Spek, 2009 ▶).

## Supplementary Material

Crystal structure: contains datablock(s) global, I. DOI: 10.1107/S160053681200195X/hp2025sup1.cif

Structure factors: contains datablock(s) I. DOI: 10.1107/S160053681200195X/hp2025Isup2.hkl

Additional supplementary materials:  crystallographic information; 3D view; checkCIF report

## Figures and Tables

**Table 1:** Hydrogen-bond geometry (Å, °)

*D*—H⋯*A*	*D*—H	H⋯*A*	*D*⋯*A*	*D*—H⋯*A*
C8—H8*A*⋯O1^i^	0.97	2.56	3.331 (4)	136

Symmetry code: (i) 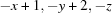.

**Fig. 1.**
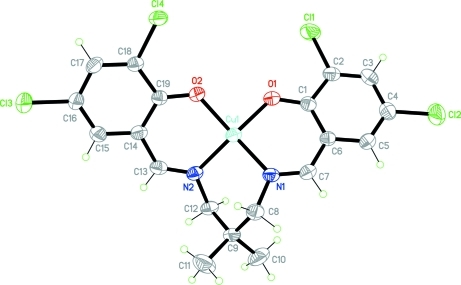
The ORTEP plot of the title compound, showing 40% probability displacement ellipsoids and the atomic numbering.

**Fig. 2.**
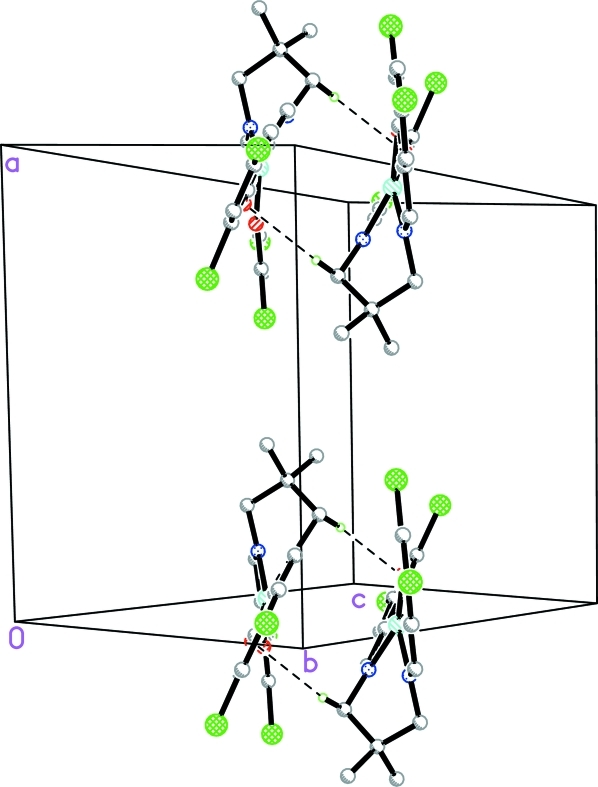
A part of the packing diagram of the title compound showing individual dimer formation through the intermolecular C—H···O intearctions (dashed lines). Only the H atoms involved in the interactions are shown.

## supplementary crystallographic information

### Comment

Schiff base complexes are one of the most important stereochemical models in transition metal coordination chemistry, with the ease of preparation and structural variations (Granovski *et al.,* 1993; Blower *et al.,* (1998). In continuation of our work on the crystal structure of Schiff base metal complexes (Kargar *et al.,* 2012; Kargar *et al.,* 2011; Ghaemi, *et al.,* (2011), we determined the X-ray structure of the title compound.

The asymmetric unit of the title compound, Fig. 1, comprises a Schiff base complex. The bond lengths (Allen *et al.,* 1987) and angles are within the normal ranges and are comparable to the related structure (Kargar *et al.,* 2012; Kargar *et al.,* 2011; Ghaemi, *et al.,* (2011).

The geometry around Cu^II^ is a distorted square-planar which is supported by the N_2_O_2_ donor atoms of the coordinated Schiff base ligand. The dihedral angle between the substituted benzene rings is 29.95 (16)°. In the crystal structure the molecules are linked together along the *b*-axis, forming individual dimers through the intermolecular C—H···O interactions (Table 1, Fig. 2). The crystal structure is further stabilized by the intermolecular π-π interaction [*Cg*1···*Cg*1^ii^ = 3.6131 (17)Å; (ii) 1 - *X*, 1- *Y*,-*Z*; *Cg*1 is the centroid of Cu(1)/O(2)/C(19)/C(14)/C(13)/N(2) ring].

### Experimental

The title compound was synthesized by adding 3,5-dichloro-salicylaldehyde-2,2-dimethyl-1, 3-propanediamine (2 mmol) to a solution of CuCl_2_. 4H_2_O (2.1 mmol) in ethanol (30 ml). The mixture was refluxed with stirring for half an hour. The resultant solution was filtered. Dark-green single crystals of the title compound suitable for *X*-ray structure determination were recrystallized from ethanol by slow evaporation of the solvents at room temperature over several days.

### Refinement

The H-atoms were included in calculated positions and treated as riding atoms: C—H = 0.93, 0.96 and 0.97 Å for CH, CH_3_ and CH_2_ H-atoms, respectively, with U_iso_ (H) = k x U_eq_(C), where k = 1.5 for CH_3_ H-atoms, and k = 1.2 for all other H-atoms.

### Crystal data

**Table:**

[Cu(C_19_H_16_Cl_4_N_2_O_2_)]	*F*(000) = 1028
*M**_r_* = 509.68	*D*_x_ = 1.625 Mg m^−^^3^
Monoclinic, *P*2_1_/*n*	Mo *K*α radiation, λ = 0.71073 Å
Hall symbol: -P 2yn	Cell parameters from 2540 reflections
*a* = 12.4002 (10) Å	θ = 2.5–27.4°
*b* = 8.4570 (7) Å	µ = 1.58 mm^−^^1^
*c* = 20.0316 (19) Å	*T* = 291 K
β = 97.278 (4)°	Block, dark-green
*V* = 2083.8 (3) Å^3^	0.25 × 0.18 × 0.09 mm
*Z* = 4	

### Data collection

**Table:**

Bruker SMART APEXII CCD area-detector diffractometer	4988 independent reflections
Radiation source: fine-focus sealed tube	2882 reflections with *I* > 2σ(*I*)
graphite	*R*_int_ = 0.060
φ and ω scans	θ_max_ = 27.9°, θ_min_ = 2.1°
Absorption correction: multi-scan (*SADABS*; Bruker, 2005)	*h* = −16→14
*T*_min_ = 0.694, *T*_max_ = 0.871	*k* = −9→11
18291 measured reflections	*l* = −26→26

### Refinement

**Table:**

Refinement on *F*^2^	Primary atom site location: structure-invariant direct methods
Least-squares matrix: full	Secondary atom site location: difference Fourier map
*R*[*F*^2^ > 2σ(*F*^2^)] = 0.047	Hydrogen site location: inferred from neighbouring sites
*wR*(*F*^2^) = 0.105	H-atom parameters constrained
*S* = 1.00	*w* = 1/[σ^2^(*F*_o_^2^) + (0.0376*P*)^2^] where *P* = (*F*_o_^2^ + 2*F*_c_^2^)/3
4988 reflections	(Δ/σ)_max_ < 0.001
255 parameters	Δρ_max_ = 0.38 e Å^−^^3^
0 restraints	Δρ_min_ = −0.36 e Å^−^^3^

### Special details

**Table:**

Geometry. All esds (except the esd in the dihedral angle between two l.s. planes) are estimated using the full covariance matrix. The cell esds are taken into account individually in the estimation of esds in distances, angles and torsion angles; correlations between esds in cell parameters are only used when they are defined by crystal symmetry. An approximate (isotropic) treatment of cell esds is used for estimating esds involving l.s. planes.
Refinement. Refinement of F^2^ against ALL reflections. The weighted R-factor wR and goodness of fit S are based on F^2^, conventional R-factors R are based on F, with F set to zero for negative F^2^. The threshold expression of F^2^ > 2sigma(F^2^) is used only for calculating R-factors(gt) etc. and is not relevant to the choice of reflections for refinement. R-factors based on F^2^ are statistically about twice as large as those based on F, and R- factors based on ALL data will be even larger.

### Fractional atomic coordinates and isotropic or equivalent isotropic displacement parameters (Å^2^)

**Table:**

	*x*	*y*	*z*	*U*_iso_*/*U*_eq_	
C1	0.5462 (3)	0.8875 (4)	0.14363 (17)	0.0370 (8)	
C2	0.6238 (3)	0.9020 (4)	0.20186 (18)	0.0428 (9)	
C3	0.6049 (3)	0.9834 (4)	0.25795 (18)	0.0468 (9)	
H3	0.6577	0.9874	0.2953	0.056*	
C4	0.5058 (3)	1.0605 (4)	0.25883 (18)	0.0486 (9)	
C5	0.4286 (3)	1.0532 (4)	0.20422 (18)	0.0430 (9)	
H5	0.3629	1.1057	0.2051	0.052*	
C6	0.4466 (3)	0.9681 (4)	0.14670 (17)	0.0365 (8)	
C7	0.3634 (3)	0.9733 (4)	0.08983 (17)	0.0398 (8)	
H7	0.3024	1.0343	0.0944	0.048*	
C8	0.2764 (3)	0.9331 (4)	−0.02093 (17)	0.0442 (9)	
H8A	0.3077	0.9657	−0.0607	0.053*	
H8B	0.2327	1.0201	−0.0078	0.053*	
C9	0.2022 (3)	0.7905 (4)	−0.03888 (18)	0.0414 (8)	
C10	0.1184 (3)	0.7758 (5)	0.0105 (2)	0.0725 (13)	
H10A	0.1548	0.7761	0.0557	0.109*	
H10B	0.0788	0.6788	0.0022	0.109*	
H10C	0.0689	0.8634	0.0045	0.109*	
C11	0.1447 (3)	0.8178 (5)	−0.1105 (2)	0.0722 (13)	
H11A	0.0918	0.7361	−0.1217	0.108*	
H11B	0.1973	0.8156	−0.1418	0.108*	
H11C	0.1091	0.9188	−0.1127	0.108*	
C12	0.2678 (2)	0.6363 (4)	−0.03537 (18)	0.0436 (9)	
H12A	0.2730	0.5953	0.0101	0.052*	
H12B	0.2283	0.5593	−0.0649	0.052*	
C13	0.4035 (3)	0.5747 (4)	−0.10521 (18)	0.0411 (9)	
H13	0.3483	0.5186	−0.1305	0.049*	
C14	0.5102 (3)	0.5676 (4)	−0.12688 (18)	0.0394 (8)	
C15	0.5173 (3)	0.4938 (4)	−0.18871 (19)	0.0490 (10)	
H15	0.4548	0.4545	−0.2138	0.059*	
C16	0.6153 (3)	0.4793 (4)	−0.21236 (18)	0.0505 (10)	
C17	0.7093 (3)	0.5351 (4)	−0.17506 (18)	0.0464 (9)	
H17	0.7761	0.5236	−0.1910	0.056*	
C18	0.7030 (2)	0.6076 (4)	−0.11442 (17)	0.0374 (8)	
C19	0.6035 (2)	0.6294 (4)	−0.08710 (17)	0.0354 (8)	
Cl1	0.74867 (8)	0.80938 (13)	0.20120 (6)	0.0735 (4)	
Cl2	0.48425 (9)	1.16671 (15)	0.33020 (6)	0.0774 (4)	
Cl3	0.62354 (9)	0.38486 (16)	−0.28877 (6)	0.0822 (4)	
Cl4	0.82198 (6)	0.67591 (10)	−0.06837 (5)	0.0476 (2)	
Cu1	0.48016 (3)	0.76529 (5)	0.01057 (2)	0.03872 (14)	
N1	0.3649 (2)	0.9025 (3)	0.03362 (14)	0.0384 (7)	
N2	0.37799 (19)	0.6517 (3)	−0.05406 (14)	0.0374 (7)	
O1	0.57082 (17)	0.8082 (3)	0.09215 (12)	0.0448 (6)	
O2	0.60372 (17)	0.6982 (3)	−0.02938 (12)	0.0431 (6)	

### Atomic displacement parameters (Å^2^)

**Table:**

	*U*^11^	*U*^22^	*U*^33^	*U*^12^	*U*^13^	*U*^23^
C1	0.0357 (19)	0.035 (2)	0.040 (2)	−0.0035 (15)	0.0030 (16)	0.0027 (16)
C2	0.0335 (19)	0.038 (2)	0.055 (2)	0.0000 (15)	−0.0015 (17)	0.0029 (18)
C3	0.048 (2)	0.045 (2)	0.045 (2)	−0.0118 (17)	−0.0026 (18)	0.0024 (18)
C4	0.050 (2)	0.054 (2)	0.042 (2)	−0.0112 (19)	0.0095 (19)	−0.0025 (18)
C5	0.040 (2)	0.045 (2)	0.045 (2)	0.0019 (17)	0.0106 (18)	0.0005 (18)
C6	0.0335 (19)	0.036 (2)	0.040 (2)	−0.0006 (15)	0.0041 (16)	0.0032 (16)
C7	0.0345 (19)	0.037 (2)	0.049 (2)	0.0021 (15)	0.0108 (17)	0.0023 (17)
C8	0.046 (2)	0.045 (2)	0.040 (2)	0.0141 (17)	−0.0017 (17)	0.0079 (17)
C9	0.0311 (18)	0.047 (2)	0.046 (2)	0.0060 (15)	0.0005 (16)	0.0039 (17)
C10	0.044 (2)	0.084 (3)	0.094 (4)	0.007 (2)	0.028 (2)	−0.004 (3)
C11	0.070 (3)	0.067 (3)	0.070 (3)	0.010 (2)	−0.027 (2)	−0.001 (2)
C12	0.0301 (18)	0.046 (2)	0.055 (2)	−0.0014 (16)	0.0085 (17)	0.0046 (18)
C13	0.0324 (19)	0.036 (2)	0.053 (2)	−0.0017 (15)	−0.0012 (17)	0.0003 (17)
C14	0.0326 (19)	0.036 (2)	0.049 (2)	0.0016 (15)	0.0036 (17)	−0.0009 (17)
C15	0.040 (2)	0.051 (2)	0.055 (3)	−0.0033 (17)	0.0005 (19)	−0.0141 (19)
C16	0.044 (2)	0.057 (2)	0.052 (3)	−0.0002 (18)	0.0116 (19)	−0.018 (2)
C17	0.039 (2)	0.048 (2)	0.056 (2)	0.0011 (17)	0.0177 (19)	−0.0066 (19)
C18	0.0329 (18)	0.0348 (19)	0.045 (2)	−0.0006 (14)	0.0066 (16)	0.0010 (16)
C19	0.0337 (19)	0.0294 (18)	0.043 (2)	0.0025 (14)	0.0034 (16)	0.0011 (16)
Cl1	0.0471 (6)	0.0767 (8)	0.0895 (9)	0.0202 (5)	−0.0185 (6)	−0.0163 (6)
Cl2	0.0730 (8)	0.1044 (9)	0.0569 (7)	−0.0119 (7)	0.0172 (6)	−0.0287 (6)
Cl3	0.0600 (7)	0.1171 (10)	0.0709 (8)	−0.0016 (6)	0.0130 (6)	−0.0493 (7)
Cl4	0.0320 (5)	0.0540 (6)	0.0568 (6)	−0.0024 (4)	0.0058 (4)	−0.0025 (5)
Cu1	0.0315 (2)	0.0416 (3)	0.0435 (3)	0.00221 (18)	0.00652 (19)	−0.0001 (2)
N1	0.0312 (15)	0.0406 (17)	0.0418 (18)	0.0036 (12)	−0.0011 (13)	0.0022 (14)
N2	0.0293 (15)	0.0356 (16)	0.0481 (18)	−0.0012 (12)	0.0086 (13)	0.0008 (14)
O1	0.0317 (13)	0.0533 (15)	0.0483 (16)	0.0058 (11)	0.0006 (11)	−0.0046 (12)
O2	0.0321 (13)	0.0509 (15)	0.0469 (15)	−0.0002 (10)	0.0073 (11)	−0.0083 (12)

### Geometric parameters (Å, °)

**Table:**

C1—O1	1.299 (4)	C11—H11A	0.9600
C1—C6	1.418 (4)	C11—H11B	0.9600
C1—C2	1.420 (4)	C11—H11C	0.9600
C2—C3	1.362 (5)	C12—N2	1.467 (4)
C2—Cl1	1.737 (3)	C12—H12A	0.9700
C3—C4	1.394 (5)	C12—H12B	0.9700
C3—H3	0.9300	C13—N2	1.287 (4)
C4—C5	1.360 (5)	C13—C14	1.444 (4)
C4—Cl2	1.737 (4)	C13—H13	0.9300
C5—C6	1.400 (4)	C14—C15	1.399 (5)
C5—H5	0.9300	C14—C19	1.419 (4)
C6—C7	1.437 (4)	C15—C16	1.365 (4)
C7—N1	1.278 (4)	C15—H15	0.9300
C7—H7	0.9300	C16—C17	1.385 (5)
C8—N1	1.470 (4)	C16—Cl3	1.741 (3)
C8—C9	1.532 (4)	C17—C18	1.372 (4)
C8—H8A	0.9700	C17—H17	0.9300
C8—H8B	0.9700	C18—C19	1.424 (4)
C9—C10	1.528 (5)	C18—Cl4	1.737 (3)
C9—C12	1.534 (4)	C19—O2	1.294 (4)
C9—C11	1.537 (5)	Cu1—O1	1.898 (2)
C10—H10A	0.9600	Cu1—O2	1.903 (2)
C10—H10B	0.9600	Cu1—N1	1.942 (3)
C10—H10C	0.9600	Cu1—N2	1.947 (3)
			
O1—C1—C6	125.2 (3)	H11A—C11—H11C	109.5
O1—C1—C2	119.6 (3)	H11B—C11—H11C	109.5
C6—C1—C2	115.2 (3)	N2—C12—C9	114.7 (3)
C3—C2—C1	123.5 (3)	N2—C12—H12A	108.6
C3—C2—Cl1	118.7 (3)	C9—C12—H12A	108.6
C1—C2—Cl1	117.8 (3)	N2—C12—H12B	108.6
C2—C3—C4	119.3 (3)	C9—C12—H12B	108.6
C2—C3—H3	120.3	H12A—C12—H12B	107.6
C4—C3—H3	120.3	N2—C13—C14	126.0 (3)
C5—C4—C3	120.0 (3)	N2—C13—H13	117.0
C5—C4—Cl2	121.3 (3)	C14—C13—H13	117.0
C3—C4—Cl2	118.7 (3)	C15—C14—C19	121.5 (3)
C4—C5—C6	121.1 (3)	C15—C14—C13	116.6 (3)
C4—C5—H5	119.5	C19—C14—C13	121.9 (3)
C6—C5—H5	119.5	C16—C15—C14	120.4 (3)
C5—C6—C1	120.8 (3)	C16—C15—H15	119.8
C5—C6—C7	117.6 (3)	C14—C15—H15	119.8
C1—C6—C7	121.5 (3)	C15—C16—C17	120.5 (3)
N1—C7—C6	126.5 (3)	C15—C16—Cl3	120.0 (3)
N1—C7—H7	116.7	C17—C16—Cl3	119.5 (3)
C6—C7—H7	116.7	C18—C17—C16	119.4 (3)
N1—C8—C9	113.9 (3)	C18—C17—H17	120.3
N1—C8—H8A	108.8	C16—C17—H17	120.3
C9—C8—H8A	108.8	C17—C18—C19	123.2 (3)
N1—C8—H8B	108.8	C17—C18—Cl4	118.6 (2)
C9—C8—H8B	108.8	C19—C18—Cl4	118.2 (3)
H8A—C8—H8B	107.7	O2—C19—C14	125.2 (3)
C10—C9—C8	110.5 (3)	O2—C19—C18	119.9 (3)
C10—C9—C12	107.6 (3)	C14—C19—C18	114.9 (3)
C8—C9—C12	111.1 (3)	O1—Cu1—O2	89.91 (10)
C10—C9—C11	110.1 (3)	O1—Cu1—N1	93.14 (11)
C8—C9—C11	107.1 (3)	O2—Cu1—N1	159.14 (11)
C12—C9—C11	110.6 (3)	O1—Cu1—N2	158.73 (10)
C9—C10—H10A	109.5	O2—Cu1—N2	93.67 (10)
C9—C10—H10B	109.5	N1—Cu1—N2	90.94 (11)
H10A—C10—H10B	109.5	C7—N1—C8	118.7 (3)
C9—C10—H10C	109.5	C7—N1—Cu1	125.7 (2)
H10A—C10—H10C	109.5	C8—N1—Cu1	115.6 (2)
H10B—C10—H10C	109.5	C13—N2—C12	119.3 (3)
C9—C11—H11A	109.5	C13—N2—Cu1	125.0 (2)
C9—C11—H11B	109.5	C12—N2—Cu1	115.0 (2)
H11A—C11—H11B	109.5	C1—O1—Cu1	127.5 (2)
C9—C11—H11C	109.5	C19—O2—Cu1	126.8 (2)
			
O1—C1—C2—C3	179.8 (3)	C15—C14—C19—C18	−1.1 (5)
C6—C1—C2—C3	1.6 (5)	C13—C14—C19—C18	177.7 (3)
O1—C1—C2—Cl1	−0.7 (4)	C17—C18—C19—O2	179.7 (3)
C6—C1—C2—Cl1	−178.9 (2)	Cl4—C18—C19—O2	−0.1 (4)
C1—C2—C3—C4	−1.5 (5)	C17—C18—C19—C14	1.1 (5)
Cl1—C2—C3—C4	179.0 (3)	Cl4—C18—C19—C14	−178.7 (2)
C2—C3—C4—C5	0.4 (5)	C6—C7—N1—C8	−174.7 (3)
C2—C3—C4—Cl2	−178.8 (3)	C6—C7—N1—Cu1	1.0 (5)
C3—C4—C5—C6	0.5 (5)	C9—C8—N1—C7	−112.5 (3)
Cl2—C4—C5—C6	179.7 (3)	C9—C8—N1—Cu1	71.4 (3)
C4—C5—C6—C1	−0.3 (5)	O1—Cu1—N1—C7	−5.6 (3)
C4—C5—C6—C7	−176.7 (3)	O2—Cu1—N1—C7	−103.6 (4)
O1—C1—C6—C5	−178.7 (3)	N2—Cu1—N1—C7	153.5 (3)
C2—C1—C6—C5	−0.6 (5)	O1—Cu1—N1—C8	170.2 (2)
O1—C1—C6—C7	−2.5 (5)	O2—Cu1—N1—C8	72.2 (4)
C2—C1—C6—C7	175.6 (3)	N2—Cu1—N1—C8	−30.7 (2)
C5—C6—C7—N1	−179.3 (3)	C14—C13—N2—C12	−174.9 (3)
C1—C6—C7—N1	4.3 (5)	C14—C13—N2—Cu1	−4.7 (5)
N1—C8—C9—C10	81.1 (4)	C9—C12—N2—C13	−118.0 (3)
N1—C8—C9—C12	−38.1 (4)	C9—C12—N2—Cu1	70.9 (3)
N1—C8—C9—C11	−159.0 (3)	O1—Cu1—N2—C13	−103.4 (3)
C10—C9—C12—N2	−153.9 (3)	O2—Cu1—N2—C13	−4.1 (3)
C8—C9—C12—N2	−33.0 (4)	N1—Cu1—N2—C13	155.5 (3)
C11—C9—C12—N2	85.8 (4)	O1—Cu1—N2—C12	67.2 (4)
N2—C13—C14—C15	−172.0 (3)	O2—Cu1—N2—C12	166.4 (2)
N2—C13—C14—C19	9.1 (5)	N1—Cu1—N2—C12	−33.9 (2)
C19—C14—C15—C16	0.1 (5)	C6—C1—O1—Cu1	−4.6 (5)
C13—C14—C15—C16	−178.7 (3)	C2—C1—O1—Cu1	177.4 (2)
C14—C15—C16—C17	1.0 (6)	O2—Cu1—O1—C1	166.7 (3)
C14—C15—C16—Cl3	179.1 (3)	N1—Cu1—O1—C1	7.4 (3)
C15—C16—C17—C18	−1.0 (6)	N2—Cu1—O1—C1	−93.3 (4)
Cl3—C16—C17—C18	−179.1 (3)	C14—C19—O2—Cu1	−10.9 (5)
C16—C17—C18—C19	−0.1 (5)	C18—C19—O2—Cu1	170.7 (2)
C16—C17—C18—Cl4	179.7 (3)	O1—Cu1—O2—C19	170.8 (3)
C15—C14—C19—O2	−179.6 (3)	N1—Cu1—O2—C19	−90.6 (4)
C13—C14—C19—O2	−0.8 (5)	N2—Cu1—O2—C19	11.8 (3)

### Hydrogen-bond geometry (Å, °)

**Table:**

*D*—H···*A*	*D*—H	H···*A*	*D*···*A*	*D*—H···*A*
C8—H8A···O1^i^	0.97	2.56	3.331 (4)	136

Symmetry codes: (i) −*x*+1, −*y*+2, −*z*.
